# Comparison between twin block appliance and mandibular advancement on clear aligners in the improvement of airway dimension: incremental versus maximum bite advancement

**DOI:** 10.3389/froh.2024.1463416

**Published:** 2024-09-03

**Authors:** Elisabetta Cretella Lombardo, Letizia Lugli, Paola Cozza, Roberta Lione, Saveria Loberto, Chiara Pavoni

**Affiliations:** ^1^ Department of Health Science, Saint Camillus International University, Rome, Italy; ^2^ Department of Health Science, UniCamillus-Saint Camillus International Medical University Rome, Rome, Italy; ^3^ Department of Health Science, Saint Camillus International University, Rome, Italy

**Keywords:** class II, aligners therapy, cephalometric analysis, sagittal airway dimensions, growing patients

## Abstract

**Objective:**

The aim of the present retrospective study was to compare the changes resulting from treatment using the MA and the TB with special regard to the oro-and naso-pharyngeal sagittal airway dimensions in subjects with dentoskeletal Class II malocclusions and positive history of Sleep Disorder Breathing (SDB) diagnosed through the Pediatric Sleep Questionnaire (PSQ).

**Materials and methods:**

This retrospective study involved 2 groups of subjects: patients treated with Twin Block (TB group: *n* = 22, 10 males, 12 females; mean age 12.0 ± 1.3 years) and patients treated with Mandibular Advancement (MA group: *n* = 23, 11 males, 12 females; mean age 12.2 ± 1.1 years). Pretreatment (T1) and posttreatment (T2) lateral cephalograms were analyzed. All patients underwent the PSQ to diagnose SDB.

**Results:**

In both treated groups there was an increase in the airways dimensions and an improvement in symptoms related SDB. The statistical comparison of the changes between T1 and T2 in the TB group showed a significant increment in upper airway size (PNS-AD2, +1.50 mm + −3.30; McNamara's upper pharynx dimension, +2.21 + −4.21) after active treatment. The MA group showed similar results during active treatment with a significant increase in both upper (PNS-AD2, +2.72 + −2.65; McNamara's upper pharynx dimension, +2.97 + −3.07) and lower (PNS-AD1, +2.17 mm + −3.54) airway size.

**Conclusions:**

Despite the different structure of these two devices and the different advancement protocols, both appliances were valuable as a suitable treatment option for Class II patients with respiratory disorders, inducing an increase of upper and lower airway size and a significant reduction in diurnal symptoms.

## Introduction

Sleep-disordered breathing (SDB) involves a range of respiratory problems during sleep, including snoring and obstructive sleep apnea (OSA) (1). These conditions are particularly concerning in pediatric populations due to their potential impact on cognitive development, growth, and overall health (2–4). Mandibular advancement devices have emerged as a promising non-invasive treatment option for managing SDB in growing patients. These devices by anterior posturing of the mandible, enlarge the upper airway and reduce the airway obstruction during sleep.

The use of orthodontic appliances for mandibular advancement in pediatric patients has been supported by several studies (5–9). A study by Villa et al. (10) demonstrated the effectiveness of oral appliances in reducing respiratory disturbances in children with OSA. Similarly, Cozza et al. (11) pointed out that functional appliances could significantly improve airway dimensions and respiratory parameters in growing patients. These findings highlight the potential benefits of mandibular advancement in managing SDB in pediatric populations (10).

In recent years, improvement in orthodontic technology have introduced clear aligners, such as Invisalign, which are primarily used for dental alignment. However, these aligners can also be designed to incorporate mandibular advancement features (12, 13). This feature allows to induce the advancement of the mandible which shifts incrementally in its proper position. This dual functionality could offer a convenient and aesthetically pleasing option for patients requiring both orthodontic treatment and SDB management.

The use of clear aligners for mandibular advancement is a relatively new area of research, in a controlled retrospective study published by Cretella et al. in 2022 (12), the authors analyzed the effects of treatment performed with the Twin Block (TB) and mandibular advancement on clear aligners (MA) in Class II subjects, concluding that both functional appliances produced a significant elongation of the mandible with an improvement in sagittal relationship, overjet, and vertical overbite values (12).

To our best knowledge, only one study, published by Yue in 2023 (14), investigated the effects of MA about the changes of upper airway morphology. In the cited study, Yue et al. performed a comparison between MA and TB appliances for the treatment of Class II patients and they concluded that both devices were effective in increasing airway dimensions (14).

Due to the widespread application for this new type of appliance and considering the impact of respiratory disorders on the health of growing patients, further studies are necessary to evaluate the effect of MA on airway dimensions and the possible positive impact on patients with breathing difficulties.

It is interesting to better understand if the different structure of this new appliance and the different advancement protocol compared to other conventional devices has an effect on its effectiveness.

Thus, the aim of the present retrospective study was to compare the changes resulting from treatment using the MA and the TB with special regard to the oro-and naso-pharyngeal sagittal airway dimensions in subjects with dentoskeletal Class II malocclusions and positive history of SDB diagnosed through the PSQ (15).

The null hypothesis tested was that both types of functional appliances were equally effective in inducing an improvement of airway size.

## Materials and methods

The study design received approval from the Ethics Committee at the Rome “Tor Vergata” Hospital, and informed consent was secured from the participants' parents for both the treatment and the potential use of their data for research purposes.

In this retrospective clinical trial, the cephalometric records of 45 patients with Class II division 1 malocclusion treated consecutively either with the TB (TB group: *n* = 22, 10 males, 12 females; mean age 12.0 ± 1.3 years), or the MA (MA group: *n* = 23, 11 males, 12 females; mean age 12.2 ± 1.1 years) were collected. Class II subjects were retrieved from the records of patients treated at the Department of Orthodontics at the Hospital of “Tor Vergata”. Participants were selected based on the following inclusion criteria: overjet ranging between 5 and 8 mm, bilateral full Class II or end-to-end molar relationships, ANB angle greater than 4°, improvement in facial profile when the lower jaw was postured forward, and cervical stage 3 in cervical vertebral maturation (CVM) at T1 (16).

Parents of all participants filled in a version of the pediatric sleep questionnaire, PSQ-SRBD Scale by Ronald Chervin (Italian version in 22 items) pre and post treatments. The questions sought information about the child's daytime symptoms (such as sleepiness, irritability, fatigue, school problems, morning headache, mouth breathing, and nasal congestion) and nighttime symptoms (including habitual snoring, apnea, restless sleep, and nightmares) (15).

Teleradiography were available at two observation points: T1, at the onset of treatment; and T2, at the conclusion of functional therapy, before orthodontic treatment with either fixed appliances or the finishing phase with additional aligners. Functional treatment ceased with a Class I molar relationship.

Study samples were selected based on skeletal maturity at the beginning of treatment, assessed using the CVM method. The CVM method can identify individual skeletal maturity in growing patients, replacing the need for hand-wrist radiographs. CVM staging was conducted by an experienced evaluator (ECL).

Demographic data for the TB and MA groups are reported in Table 1. All patients were treated by two skilled orthodontist, whose experience in managing the two functional appliances was comparable in terms of years of practice and the number of patients treated with functional devices.

**Table 1:** Demographics of the TB and MA groups.

	Age at T1, y		Age at T2, y		T1-T2, y	
	Mean	SD	Mean	SD	Mean	SD
TB Group (*n* = 22; 12 f, 10 m)	12.0	1.3	13.8	1.3	1.8	0.5
MA Group (*n* = 23; 12 f, 11 m)	12.2	1.1	13.7	1.2	1.5	0.6
*P*-value	0.5797		0.7898		0.0761	

y, indicates years; SD, standard deviation; f, female; m, male.

### Treatment protocol

**Figure 1:**
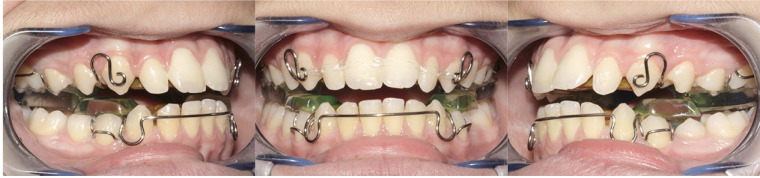
Frontal and lateral views of a twin block (TB) appliance.

Patients in the TB group were treated with a TB device designed according to Clark's original concept (Figure 1). The appliance consisted of maxillary and mandibular plates fitting against the teeth, alveolus, and other supporting structures. Delta or Adams clasps were constructed on both sides to anchor the upper plate to the first permanent molars, and 0.030-inch ball clasps (or arrow clasps) were placed in the anterior interproximal spaces. The precise arrangement of the clasps depended on the state of dentition at the time of TB construction. In the mandibular arch, Clark suggested placing ball hooks between the canines and incisors.

For each patient, the construction bite was created in a single step, with maximum bite advancement. The construction bite allowed for a 5–7 mm vertical opening in the area of the posterior bite blocks. An important advantage of the twin block is the ability to guide the vertical eruption of posterior teeth through selective removal of acrylic during therapy. In hypodivergent patients with short lower anterior facial height and/or a deep curve of Spee, the acrylic on the posterior area of the upper bite block was trimmed to encourage the eruption of the lower posterior teeth. All subjects in this study were advised to wear the device full-time for a minimum of 22 h a day (excluding meals and sports) until the end of therapy (17).

**Figure 2:**
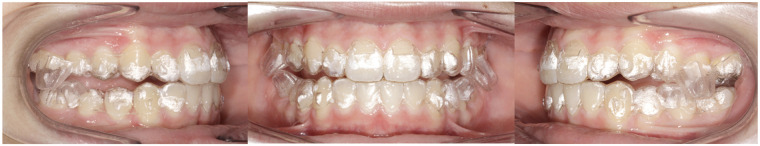
Frontal and lateral views of a mandibular advancement (MA) appliance.

Patients in the MA group were treated with the Mandibular Advancement (MA) appliance (Figure 2). The aligners feature precision wings made from the patented SmartTrack® material, located between the premolars and first molars, to hold the mandible in a forward position. With the MA appliance, mandibular advancement was not programmed in a single step but incrementally. While the aligners worked on orthopedic correction, they also aligned and leveled the teeth simultaneously. An initial pre-MA phase was automatically applied in specific situations (deep bite >7 mm, molar rotation >20°, Class II division 2, and cross-bite) to allow for wing placement or the first advancement. After mandibular advancement, a transitional phase was planned to hold the mandible in the advanced position while awaiting the delivery of standard or additional aligners. As with regular aligner treatment, patients were instructed to wear the aligners for a minimum of 22 h a day, removing them only to eat, drink, brush, and floss. Aligners were changed weekly (12).

### Cephalometric analysis

All lateral cephalograms of each patient were manually traced in a single session. The tracings were performed by one investigator, and the accuracy of landmark locations and anatomical outlines was verified by a second investigator. Any discrepancies in landmark placement were resolved through mutual agreement. A customized digitization regimen (Viewbox, version 4.0, dHAL Software, Kifissia, Greece) was created and utilized for the cephalometric evaluation.

**Figure 3:**
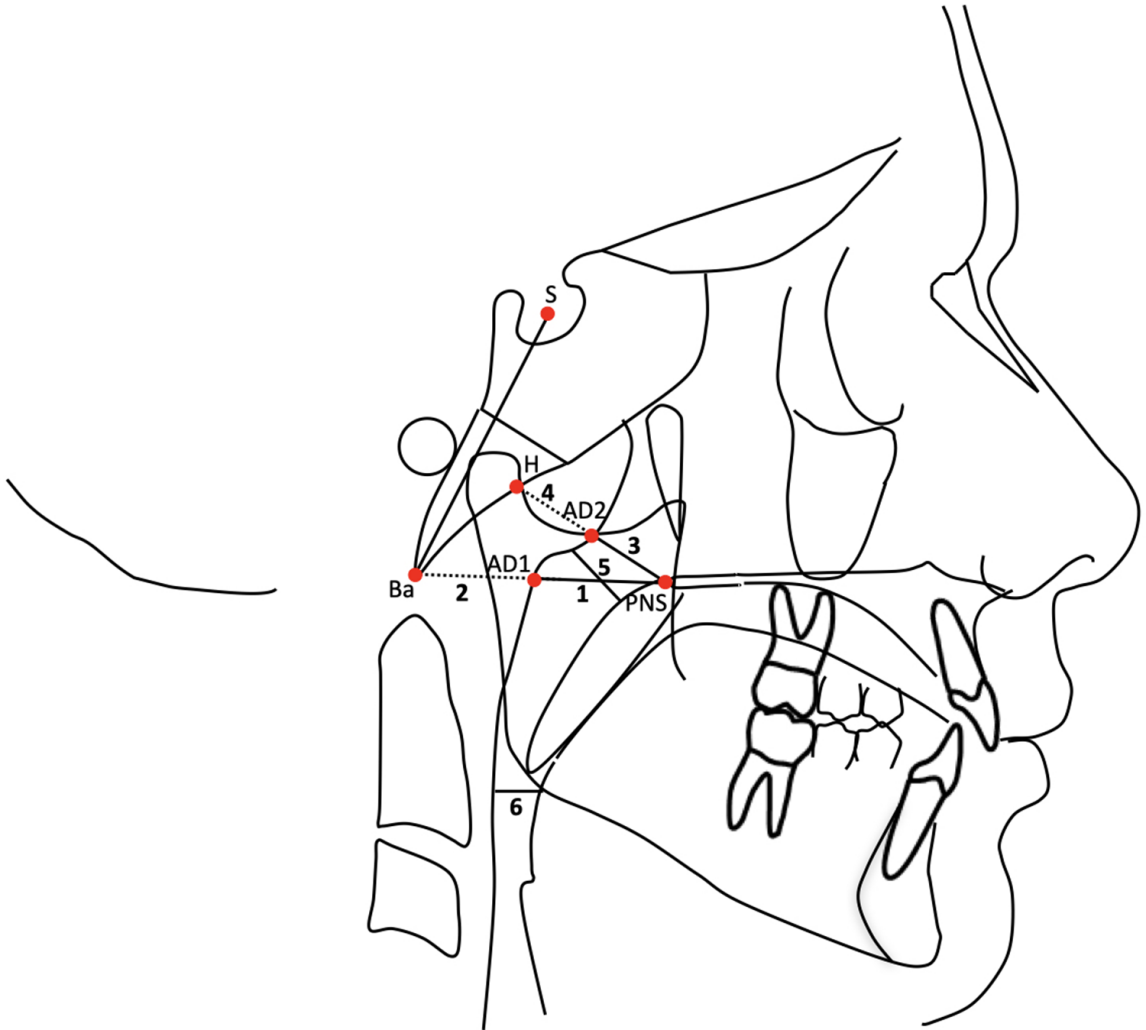
Cephalometric measurements for the analysis of airway dimensions.

The cephalometric measurements used were (Figure 3) (18, 19):

1) PNS-AD1: lower airway dimension; the distance between the Posterior Nasal Spine (PNS) and the nearest adenoid tissue measured through the PNS-Ba line (AD1).
2) AD1-Ba: lower adenoid size; defined as the soft tissue thickness at the posterior nasopharynx wall through the PNS-Ba line.
3) PNS-AD2: upper airway dimension; the distance between the PNS and the nearest adenoid tissue measured through a perpendicular line to S-Ba from PNS (AD2).
4) AD2-H: upper adenoid size; defined as the soft tissue thickness at the posterior nasopharynx wall through the PNS-H line (H, Hormion, located at the intersection between the perpendicular line to S-Ba from PNS and the cranial base).
5) McNamara's upper pharynx dimension: the minimum distance between the upper soft palate and the nearest point on the posterior pharynx wall.
6) McNamara's lower pharynx dimension: the minimum distance between the point where the posterior tongue contour crosses the mandible and the nearest point on the posterior pharynx wall.

### Statistical analysis

The Fisher Exact test was used to compare gender distribution. Descriptive statistics and statistical comparisons between the TB and MA groups at T1 (starting forms) and for the T2-T1 inter and intra-group changes were assessed using Independent samples *t*-test, with the *P*-value set at *P* ≤ 0.05.

### Method error

Fifteen lateral cephalograms, randomly selected, were re-measured after a washout period of 2 weeks by the same operator (ECL). Intraobserver reproducibility was assessed with the intraclass correlation coefficient (ICC), while the method of moments' estimator (MME) was applied for assessing random error.

## Results

The demographic data of the treated and the control groups are reported in Table 1. No significant between-group differences were found either for chronologic age at T1 (*P* = 0.5797), at T2 (*P* = 0.7898) and for gender distribution (*P* = 1.000). The duration of treatment was similar for both groups (*P* = 0.0761).

For each patient included in the present investigation, the Pediatric Sleep Questionnaire (PSQ) indicated a positive result for sleep-related breathing disorders before treatment.

The analysis of the starting forms showed no significant differences between groups for any airway measurements (Table 2).

**Table 2:** Descriptive statistics and statistical comparisons (independent-samples *t*-tests) of the starting forms (cephalometric values at T1).

Variables	TB (*n*: 22)		MA (*n*: 23)		Difference	*P*-value	95% CI of the difference	
	Mean	SD	Mean	SD			Lower	Upper
AD1-Ba	19.24	3.12	20.07	3.77	0.83	0.427	−1.19	2.85
AD2-H	14.94	1.87	15.00	3.52	0.06	0.944	−1.58	1.70
McNamara's lower pharynx	9.77	2.00	10.94	2.02	1.17	0.058	0.00	2.34
PNS-AD1	20.47	4.08	21.43	3.64	0.96	0.409	−1.30	3.22
PNS-AD2	15.70	2.98	15.69	3.17	−0.01	0.991	−1.81	1.79
McNamara's upper pharynx	10.20	2.80	11.47	1.82	1.27	0.077	−0.12	2.66

SD, standard deviation; CI, confidence of interval; *P* < 0.05.

The statistical comparison of the changes between T1 and T2 in the TB group (Table 3) showed a significant increment in upper airway size after active treatment (PNS-AD2, upper airway dimension; distance between the PNS and the nearest adenoid tissue measured through a perpendicular line to S-Ba from PNS: +1.50 mm + −3.30; McNamara's upper pharynx dimension, the minimum distance between the upper soft palate and the nearest point on the posterior pharynx wall: +2.21 + −4.21), no significant differences were found in lower airway size.

**Table 3:** Descriptive statistics and statistical comparisons (independent-samples *t*-test) of the T2-T1 changes in the TB.

Variables	T1 (*n* = 22)		T2 (*n* = 22)		Difference	*P*-value	95% CI of the difference	
	Mean	SD	Mean	SD			Lower	Upper
AD1-Ba	19.24	3.12	18.45	3.96	−0.79	0.355	−2.371	0.791
AD2-H	14.94	1.87	13.39	4.03	−1.55	0.068	−2.943	−0.157
McNamara's lower pharynx	9.77	2.00	10.43	3.92	0.66	0.473	−0.720	2.040
PNS-AD1	20.47	4.08	21.58	6.00	1.11	0.416	−1.165	3.385
PNS-AD2	15.70	2.98	17.20	3.67	1.50	0.045*	0.018	2.982
McNamara's upper pharynx	10.20	2.80	12.41	5.06	2.21	0.022*	0.397	4.023

SD, standard deviation; CI, confidence of interval; *P* < 0.05.

Asterisks are used to indicate the level of significance associated with *P*-values ​​in the results of statistical analyses; one asterisk (*) indicates a *P* < 0.05.

**Figure 4:**
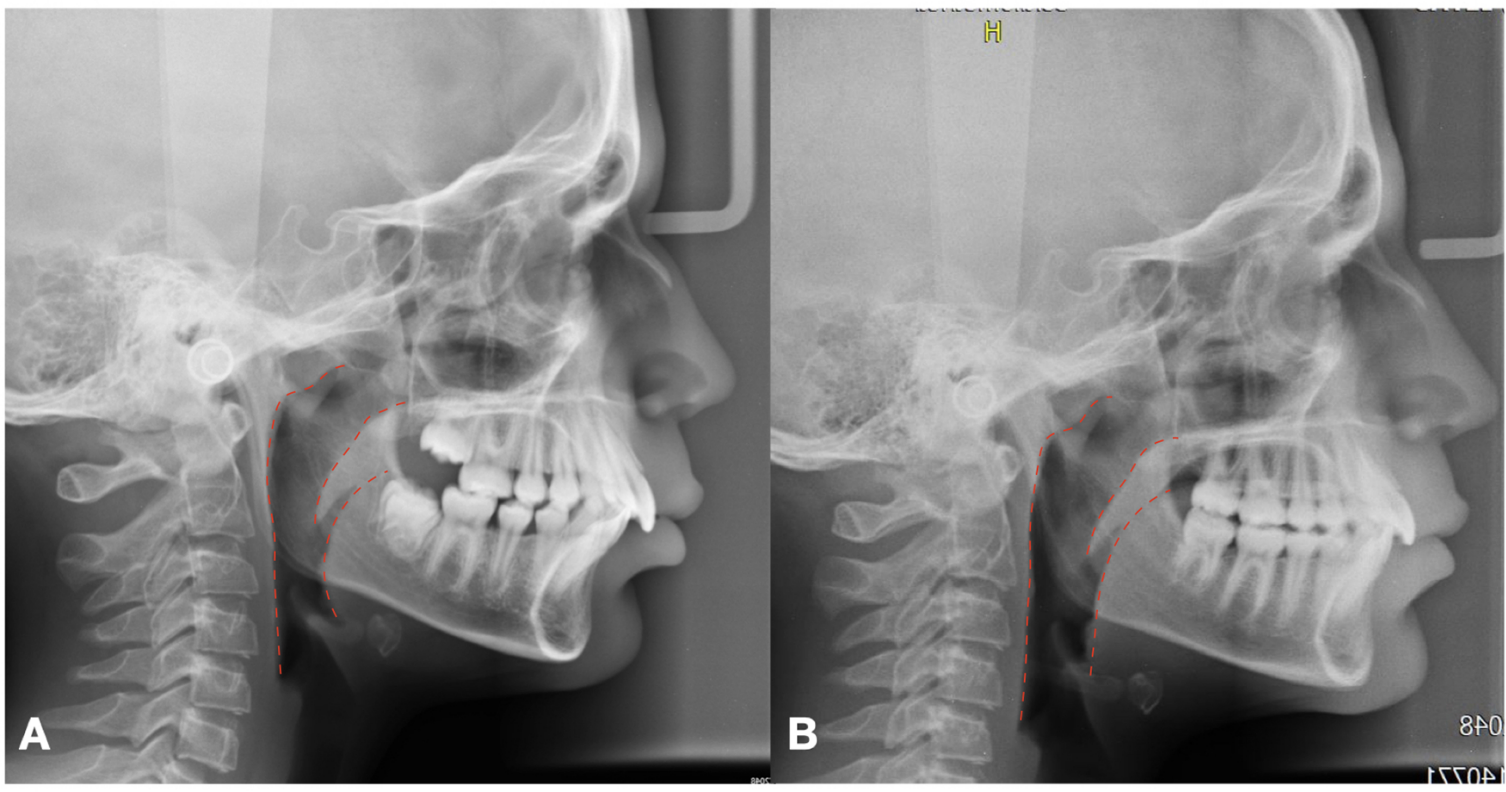
Increase of airway dimensions on Pre **(A)** and post **(B)** treatment lateral cephalogram.

The MA group showed similar results during active treatment (T1-T2; Table 4), with a significant increase in both upper (PNS-AD2, upper airway dimension; distance between the PNS and the nearest adenoid tissue measured through a perpendicular line to S-Ba from PNS:+2.72 + −2.65; McNamara's upper pharynx dimension, the minimum distance between the upper soft palate and the nearest point on the posterior pharynx wall: +2.97 + −3.07) and lower (PNS-AD1, lower airway dimension; distance between the PNS and the nearest adenoid tissue measured through the PNS-Ba line: +2.17 mm + −3.54) airway size (Figure 4).

**Table 4:** Descriptive statistics and statistical comparisons (independent-samples *t*-test) of the T2–T1 changes in the MA.

Variables	T1 (*n* = 23)		T2 (*n* = 23)		Difference	*P*-value	95% CI of the difference	
	Mean	SD	Mean	SD			Lower	Upper
AD1-Ba	20.07	3.77	18.99	3.86	−1.07	0.110	−2.730	0.570
AD2-H	15.00	3.52	13.7	3.34	−1.3	0.206	−0.739	3.339
McNamara's lower pharynx	10.94	2.02	11.63	2.47	0.70	0.180	−0.286	1.666
PNS-AD1	21.43	3.64	23.60	3.79	2.17	0.008**	0.563	3.777
PNS-AD2	15.69	3.17	18.40	3.30	2.72	0.000***	1.311	4.109
McNamara's upper pharynx	11.47	1.82	14.43	3.22	2.97	0.000***	1.829	4.091

SD, standard deviation; CI, confidence of interval; *P* < 0.05.

Asterisks are used to indicate the level of significance associated with *P*-values ​​in the results of statistical analyses; two asterisks (**) indicate a *P* < 0.01; three asterisks (***) indicate a *P* < 0.001.

The statistical comparison of T2-T1 changes between the TB and MA groups showed no statistically significant differences for any airway analyzed measurements (Table 5).

**Table 5:** Descriptive statistics and statistical comparisons (independent-samples *t*-test) of the T2-T1 changes in the TB vs. the MA.

Variables	TB (*n*: 22)		MA (*n*: 23)		Difference	*P*-value	95% CI of the difference	
	Mean	SD	Mean	SD			Lower	Upper
AD1-Ba	−0.88	4.06	−1.07	3.10	−0.19	0.86	−2.31	1.93
AD2-H	−0.31	3.64	−1.30	2.63	−0.99	0.299	−2.89	0.91
McNamara's lower pharynx	1.25	4.59	0.70	2.41	−0.55	0.615	−2.71	1.61
PNS-AD1	1.46	5.66	1.27	3.05	−0.19	0.888	−2.86	2.48
PNS-AD2	1.36	4.18	2.34	2.81	0.98	0.359	−1.11	3.07
McNamara's upper pharynx	2.19	3.42	3.19	2.33	1.00	0.256	−0.72	2.72

SD, standard deviation; CI, confidence of interval; *P* < 0.05.

At the end of the treatment, the children's parents again completed the same questionnaire and a significant reduction in diurnal symptoms was observed in all the treated patients.

## Discussion

Functional appliances are orthodontic devices designed to modify the position of the mandible and stimulate the growth. Nowadays, mandibular advancement is an orthodontic practice used not only to improve the sagittal skeletal relationship of growing patients, but also to treat respiratory disorders. Indeed, Mandibular advancement with functional appliances aims to reduce the obstruction of the upper airways, improving airflow during nocturnal breathing.

Several studies in literature analyzed the effects of different devices for mandibular advancement in increasing airway dimensions. For the existing literature, functional appliances have stronger scientific evidence supporting their effectiveness, with numerous papers confirming their efficacy (20–24).

On the contrary, very poor is the literature supporting the positive effects induced by Mandibular advancement with Clear Aligners on the sagittal airway dimension in growing patients (14).

Therefore, the objective of the present research was to compare the effects resulting from treatment with the MA and the TB given the differences in terms of material and advancement protocols. The TB appliance induces a maximum bite advancement in a single step, while with the MA the jaw shift incrementally forward. In the literature there are very conflicting opinions on which is the most effective advancement protocol. Nowadays there is greater scientific evidence in favor of incremental mandibular advancement in terms of mandibular response and increase in mandibular length. It is interesting to note that on the basis of our results, the different advancement protocol applied by these two devices did not produce differences in the improvement of airways dimension and SDB symptoms.

The results of the present study concluded that both TB and MA were able to induce an increasing of airway dimension. In particular TB group showed an improvement of the airway size mainly located in the upper adenoid tissue whereas MA patients showed a reduction of adenoid tissue both at upper and lower level.

As reported in literature, the reduction of adenoid tissue through the use of functional appliance occurs primarily by improving the patency of the upper airway. This enhancement can decrease the hypertrophy of adenoid tissue caused by chronic obstruction. The main mechanism behind this phenomenon lies in the ability of functional appliances to advance the mandible and tongue, thereby increasing the airway space and reducing the likelihood of airway collapse during sleep (25, 26).

Mandibular advancement can result in a significant increase in the volume of the upper airway, including both the upper and lower oropharynx. However, the effect is often more pronounced in the superior adenoid tissue, which is directly involved in the obstruction of the upper airway. This is due to the increased airflow and the reduction of negative pressure that contributes to adenoid hypertrophy (25, 26).

Moreover, our results could be explained by a better management of inflammatory response of adenoid tissue performed with incremental mandibular advancement compared to single-phase maximum protrusion. Incremental advancement allows for gradual adaptation of the tissues, reducing the risk of excessive inflammation (27, 28).

According to our research, a study conducted by Iwasaki et al. demonstrated that the use of functional appliances can significantly increase the dimensions of the upper airways in patients with OSA. The results showed an increase in pharyngeal volume and a reduction in OSA symptoms, confirming the effectiveness of functional appliances in improving respiratory function (29–31).

A further study by Pavoni et al, published in 2017 found that the treatment with functional appliances produced significant favorable changes during active treatment in the oro- and nasopharyngeal sagittal airway dimensions in subjects with dentoskeletal Class II subjects when compared with untreated controls. The favorable changes obtained during T1-T2 interval were maintained in the long-term observation after puberty (22).

Aligners with an integrated mandibular advancement mechanism have been recently introduced, combining the benefits of invisible orthodontics with mandibular advancement to improve airways.

Similar to our study, the paper published by Yue et al. in 2023 compared the effects of Invisalign with mandibular advancement and Twin Block appliance. The cited study evaluates and compares the improvement of upper airway morphology and hyoid bone position in children with Class II mandibular retrusion treated with these two types of appliances, by means of cone beam computed tomography (CBCT) (14).

The authors, according to our results, found that both MA and TB appliances effectively improved the structural narrowness of the upper airway and reduced respiratory resistance, thus improving breath quality with a better comfort and adherence to treatment by patients with Invisalign system. However, in the study performed by Yue, MA showed more effectiveness in improving the narrowest part of the hypopharynx compared to TB (14).

In conclusion, mandibular advancement represents an effective strategy to increase airway dimensions with solid scientific base supporting the effectiveness of functional appliances. It is important to consider that studies on Invisalign MA are still limited and further research is needed to confirm its long-term effectiveness. Mandibular advancement with aligners offers advantages in terms of comfort and treatment adherence, but the high cost can represent a barrier for some patients.

The choice of the most appropriate device depends on the individual needs of patients, considering factors such as the severity of OSA, aesthetics, comfort, and costs.

It is fundamental to carefully evaluate these variables to provide the appropriate treatment for patients.

A primary limitation of this study is the small sample size, which should be increased in future research. Additionally, the absence of a control group and the short-term nature of the study are significant constraints. Having a control group is crucial as it allows for comparison against a baseline, thereby enhancing the validity of the results by isolating the effect of the intervention. Evaluating the long-term stability of the findings is also important to determine the persistence and durability of the observed effects, which would provide a more comprehensive understanding of the intervention's impact over time. Future studies will aim to overcome these limitations to validate the current results and assess their long-term stability.

## Conclusions

Functional therapy performed with TB and MA produce the following:

- Despite the different structure of the analyzed devices and the different advancement protocols, both appliances were suitable treatment options for Class II patients with respiratory disorders, inducing an increase of the airway dimension and a significant reduction in diurnal symptoms in all patients;
- Patients treated with the TB appliance showed a significant improvement of airway dimension after active treatment mainly located in the upper adenoid tissue whereas MA patients showed a reduction of adenoid tissue both at upper and lower level probably as a consequence of a better adaptation of the tissue to incremental advancement.

## Data Availability

The raw data supporting the conclusions of this article will be made available by the authors, without undue reservation.
