# Supplementary material for: Comparison between twin block appliance and mandibular advancement on clear aligners in the improvement of airway dimension: incremental versus maximum bite advancement
Source: Front Oral Health. 2024 Sep 3;5:1463416. doi: 10.3389/froh.2024.1463416 (PMC11405374; doi:10.3389/froh.2024.1463416)
Supplement: Supplementary file 1 [file Datasheet1.pdf]

Pediatric Sleep Questionnaire: Sleep-Disordered Breathing Subscale

Nome del bambino: ________________________________

Persona intervistata: ______________________ **Data:** ____/____/____

Si prega di rispondere alle seguenti domande riguardanti le abitudini del tuo bambino durante il sonno o la veglia. Le domande si riferiscono alle abitudini che ha avuto il tuo bambino durante l'ultimo mese. Cerchia la risposta corretta rispondendo con SI, NO, Non So (NS).

1. MENTRE DORME, IL TUO BAMBINO:
   - Russa più della metà del tempo?………………………………………….……… SI NO NS A2
   - Russa sempre? ……………………………………………………………………SI NO NS A3
   - Russa forte? ………………………………………………………………… SI NO NS A4
   - Ha un respiro pesante o rumoroso? ………………………………………..…… SI NO NS A5
   - Ha difficoltà a respirare o si sforza a respirare? ……………………..……… SI NO NS A6
2. HAI MAI VISTO IL TUO BAMBINO FARE DELLE PAUSE RESPIRATORIE DURANTE LA NOTTE? ……………………………………………………… SI NO NS A7
3. IL TUO BAMBINO:
   - Tende a respirare con la bocca aperta durante il giorno?……………………… SI NO NS A24
   - Al mattino, quando si sveglia, ha la bocca secca? . ……………………………..SI NO NS A25
   - Occasionalmente bagna il letto? ………………………………………………… SI NO NS A32
4. IL TUO BAMBINO:
   - Si sveglia sentendosi poco riposato al mattino? ……………………………… SI NO NS B1
   - Ha problemi di sonnolenza durante il giorno? ……………………….……….....SI NO NS B2
5. GLI INSEGNANTI HANNO FATTO NOTARE CHE IL TUO BAMBINO APPARE ASSONNATO DURANTE IL GIORNO? ……………………………...SI NO NS B4
6. È DIFFICILE SVEGLIARE IL TUO BAMBINO AL MATTINO? ………………..…..SI NO NS B6
7. IL TUO BAMBINO SI SVEGLIA COL MAL DI TESTA AL MATTINO?……… SI NO NS B7
8. HA SMESSO DI CRESCERE REGOLARMENTE IN UN CERTO PERIODO DELLA SUA VITA……………………………………………………….… SI NO NS B9
9. IL TUO BAMBINO È IN SOVRAPPESO? ………………………………………….SI NO NS B22
10. IL TUO BAMBINO SPESSO:
    - Non sembra ascoltare quando gli si parla direttamente? …………………………. SI NO NS C3
    - Ha difficoltà ad organizzare compiti e attività?. ……………………..………….... SI NO NS C5
    - È facilmente distratto da stimoli esterni?. …………………………..……….... SI NO NS C8
    - Si agita con le mani o con i piedi o appare irrequieto quando sta seduto?.........................…………………………………..... SI NO NS C10
    - È sempre in movimento o agisce come se fosse ipercinetico?. …………………. SI NO NS C14
    - Interrompe o si intromette fra gli altri (si inserisce dentro conversazioni o giochi)?………………………………………SI NO NS C18

GRAZIE!

© Regents of the University of Michigan 2007

Traduzione Italiana in accordo con l'Autore: Ranieri Salvatore e Paola Cozza, Università degli Studi di Roma "Tor Vergata"2015
